# Analysis of the effectiveness of improving the testing rate of pathogenicity specimens prior to the use of antibiotics in Shandong Province, 2014–2023

**DOI:** 10.3389/fpubh.2025.1645769

**Published:** 2025-12-16

**Authors:** Zhiyuan Chen, Jian Sun, Weiguang Li

**Affiliations:** Department of Infection Control, Provincial Hospital of Shandong First Medical University, Jinan, Shandong, China

**Keywords:** antibiotics, pathogenesis, specimen testing, information system, management

## Abstract

**Objective:**

To analyze the trend of pathogenic specimen detection rate before antibiotic treatment in inpatients in Shandong Province from 2014 to 2023 and to provide a scientific basis for optimizing antibiotic management.

**Methods:**

The time of antibiotic use and the time of pathogenic specimen collection for inpatients in each hospital were collected through the hospital information system, the test information system, and the mobile care system. The process data of specimen detection rate were calculated according to different time points: the pre-antibiotic treatment pathogenic detection rate, the detection rate within 12 h after antibiotic treatment, the detection rate within 24 h, and the detection rate before discharge.

**Results:**

The detection rate of pre-antibiotic therapy pathogenicity specimens among inpatients in Shandong Province from 2014 to 2023 showed an overall increasing trend from 7.67 to 38.38%, while the pre-discharge detection rate increased from 32.84 to 67.48%; both differences were statistically significant (*p* < 0.01). The percentage of pathogenic samples sent for testing 0–12 h after antimicrobial drug treatment decreased from 26.74 to 25.10%, and the percentage sent 12–24 h after treatment decreased from 23.96 to 12.18%.

**Conclusion:**

The detection rate of pre-antibiotic therapy pathogenicity specimens among inpatients in Shandong Province showed an increasing trend from 2014 to 2023, but the number of people tested only 12 h after antibiotic therapy is still high and so needs to be focused on. This could be achieved by improving hospital information systems and increasing training.

## 1 Introduction

Due to the wide and sometimes unnecessary application of antibiotics bacterial resistance is becoming a global issue, and research data show that, in recent years, there have been more than 1 million deaths annually due to drug-resistant bacterial infections worldwide (1). According to the World Health Organisation, if the drug-resistant situation cannot be effectively controlled, the number of deaths per year will rise to 10 million in 2050. How exactly to curb bacterial drug resistance and to ensure rational application of antibiotics has become an important issue in the field of global public health (2, 3). The importance of microbial pathogenicity testing has been increasing around the world, and many countries have introduced a series of testing guidelines. For example, the Centres for Disease Control (CDC) for the United States, Nigeria, and other countries have emphasized that clinicians should prescribe antibiotics based on the test report and improve the level of test delivery (4, 5). Therefore, the rational use of antibiotics is crucial, and the choice of antibiotics should in principle be based on the results of bacterial drug sensitivity tests. These tests require continuous improvement in the rate of microbiological specimen delivery to achieve the rational use of antibiotics according to the type of bacteria.

In recent years, to achieve the above goals, China’s health administration introduced the 2015 Edition of the Hospital Infection Management Quality Control Indicators and subsequently designated “increasing the rate of pre-treatment antimicrobial drug delivery for inpatients” as one of the top 10 national healthcare quality and safety improvement goals for 2021 and 2022. In 2022, a series of policy measures such as the Notice on Further Strengthening the Management of Clinical Application of Antimicrobial Drugs to Curb Bacterial Drug Resistance and the National Action Plan for Curbing Microbial Drug Resistance (2022–2025) were issued, emphasizing the strengthening of hospital informatization, standardization of diagnosis and treatment of infections, enhancement of pathogenetic diagnostic capacity, and advancement of the effective management of antimicrobial drugs (6). In 2023, another series of policies and measures were introduced to further promote the improvement of in-patient antimicrobial drug treatment and increase the rate of pre-treatment pathogenetic delivery of in-patient specimens. In 2023, a letter was issued on further promoting the improvement of the inpatient antimicrobial pre-treatment pathogenetic delivery rate, requiring medical institutions to attach great importance to the work of inpatient antimicrobial pre-treatment pathogenetic delivery so as to effectively reduce the detection rate of multi-drug-resistant bacteria (7). Early detection of causative pathogens and targeted antimicrobial therapy prevent the emergence of multidrug-resistant organisms (MDROs) through empirical treatment, effectively reducing MDRO infection rates (8).

However, the traditional data collection method calculates the detection rate of pathogenic specimens without considering the specific time of collection or applying temporal limits to the indicators, which can lead to biased statistical results. In this study, we collected data on the process of calculating the detection rate of pre-antibiotic therapy pathogenicity specimens from 2014 to 2023, including the time of antibiotic administration and the time of specimen delivery, analyzed the impact of different collection methods on the detection rate of pathogenicity specimens, and accurately analyzed the detection rate of pre-antibiotic therapy pathogenicity specimens. This provides a scientific basis for the continuous improvement of the detection rate of pathogenicity specimens, the rational use of antibiotics, and the reduction of drug-resistant bacteria.

### 1.1 Objects and methods

#### 1.1.1 Objects of the study

Using data from the Shandong Province hospital infection management monitoring network (the administrative level of which includes provincial-, municipal-, and county-level hospitals, and the nature of hospitals include general and specialist hospitals, with specialist hospitals focusing on areas such as oncology, Chinese medicine, ophthalmology, obstetrics and gynecology.), the processing data of antibiotic treatment pathogenicity specimen tests were calculated as the object of the study by collecting the elements of antibiotic dosing time and pathogenicity specimen testing time from 2014 to 2023 for the network units with an information technology foundation. Inclusion criteria were patients using antibiotics administered systemically for therapeutic purposes. Exclusion criteria were patients with topical or prophylactic use of antibiotics.

#### 1.1.2 Research indicators

The indicators of this study include processing data such as the detection rate of pathogenicity specimens before antibiotic treatment, the detection rate of pathogenicity specimens within 12 and 24 h of antibiotic treatment, and the detection rate of pathogenicity specimens before discharge, in which the detection rate of pre-discharge pathogenicity specimens is the data collected by traditional methods.

Calculation Formula: Denominator: The number of inpatients treated with antibiotics during the same period. Numerator: The number of pathogenic specimens detected before antibiotic treatment.

The therapeutic use of antibiotics in this indicator refers to the systemic administration of antibiotics for ‘therapeutic’ purposes by oral, rectal, sublingual, injectable, subcutaneous, intramuscular, intravenous, or intravenous drip routes of administration. Specimen testing items include microbiological culture and drug sensitivity tests, microscopic examination, immunological detection, and molecular rapid diagnosis. Therapeutic medication is indicated solely for patients with confirmed or highly suspected infections caused by pathogenic microorganisms such as bacteria or fungi. Prompt pathogen identification is essential: where feasible, pathogen testing (e.g., blood cultures or sputum cultures) should be conducted expeditiously, with antimicrobial selection guided by susceptibility testing results. The objective is to control and eradicate infection, necessitating prolonged treatment courses adjusted according to clinical response. Prophylactic antimicrobial therapy is indicated for specific high-risk populations to prevent infection by particular pathogens, but indications must be strictly limited to, for example, perioperative settings, non-surgical patients, or special procedures. The objective is to reduce the probability of infection occurrence, with a short treatment duration and strict restrictions on indications and target populations. These guidelines are implemented in accordance with the Chinese government’s Guidelines for the Clinical Application of Antimicrobial Agents (2015 Edition).

#### 1.1.3 Data collection

Data collection in this study was divided into two approaches: traditional collection and information collection. The traditional method focused on the pre-discharge specimen submission rate, whereas the information collection method measured the rate of pathogenic specimens submitted prior to antimicrobial drug treatment. The calculation of the indicator requires that the collection time of pathogenetic specimens should be earlier than the time of antibiotic use, and the accurate testing and antibiotic use time must be obtained, so the data source is preferred to the time of collection of pathogenetic specimens and the time of the first antibiotic use. In the hospital information system (HIS), specimens whose antibiotics are used for ‘therapeutic’ and systemic routes of administration are extracted; in the laboratory information system (LIS), specimens including microbial culture and drug sensitivity tests, microscopic examinations, immunological tests, molecular rapid diagnosis, and related marker tests are extracted. Microbial culture and susceptibility testing included Common bacteria, anaerobic bacteria, microaerophiles, fungi, mycobacteria, and mycoplasma. Microscopic examination included sterile body fluid (centrifuged) smear staining for bacterial examination, fungal smear examination, acid-fast staining, Gram staining, silver staining, gonococcal Gram staining, and cryptococcal India ink staining. Immunological testing included *Streptococcus pneumoniae* antigen, Legionella antigen/antibody detection, and special pathogens. Antigen–antibody testing included Mycoplasma, Chlamydia, Rickettsia, Spirochetes, Cryptococcus, *Clostridium difficile*, Brucella, Salmonella, Shigella, Culture-based serological agglutination, *Treponema pallidum* particle agglutination, TPPA+ rapid plasma reagin ring card test (RPR), G test, and GM test. Molecular Rapid Diagnostic Testing Items included Polymerase Chain Reaction (PCR), Mass Spectrometry (MS), and Gene Sequencing. The Personal Digital Assistant (PDA) used accurately extracts the time of collection of pathogenicity specimens, the time of the first treatment of antibiotic medication, and the time of collection and testing of pathogenicity specimens; the hospital infection management information system interfaces with the relevant healthcare business systems to obtain accurate data on pathogenicity testing and antibiotic use and conducts data sorting and standardization.

#### 1.1.4 Intervention measures

Establish a multi-departmental collaboration mechanism, clarify the responsibilities of each department, refine the working system of the hospital’s special group on the antimicrobial pre-treatment pathogenicity delivery rate, and convene regular working meetings.

Maintain the information monitoring system; streamline and improve pathogenicity testing protocols and specimen submission methods; and implement a submission reminder pop-up.

Conduct Multi-Disciplinary Treatment (MDT) discussions and specialized training, providing effective clinical guidance and timely feedback.

Incorporate these measures into the hospital’s quality management performance assessment, linking them to performance-based rewards and penalties to create an incentive and constraint mechanism.1.5 Statistical analysis.

#### 1.1.5 Statistical analysis

The data were analyzed using SPSS26.0 software, and the counting information was expressed as the number of cases or percentage, which was statistically analyzed using the *χ*^2^ test with a test level of *α* = 0.05 (two-sided).

## 2 Results

### 2.1 Distribution of hospitals

**Figure 1:**
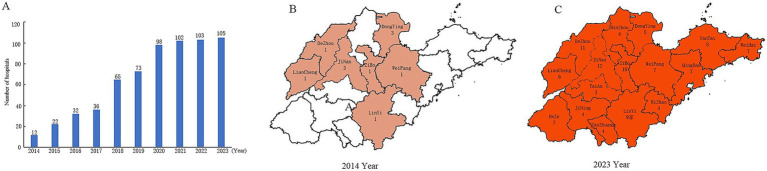
Number of hospitals reporting data on pathology delivery rates in Shandong Province, 2014-2023. **(A)** Changes in the number of hospitals, **(B)** Hospital coverage in 2014, **(C)** Hospital coverage in 2023.

After the comprehensive interventions, the number of hospital units collecting data on the detection rate of antibiotic treatment pathogenicity specimens in Shandong Province increased year by year from 2014 to 2023, with the total number of hospitals increasing from 12 to 105 (Figure 1A) and the coverage area expanding from 7 municipalities to 16 municipalities in the province (Figures 1B, C).

### 2.2 Overall changes in the detection rate of antibiotic therapy pathogenicity specimens

**Figure 2:**
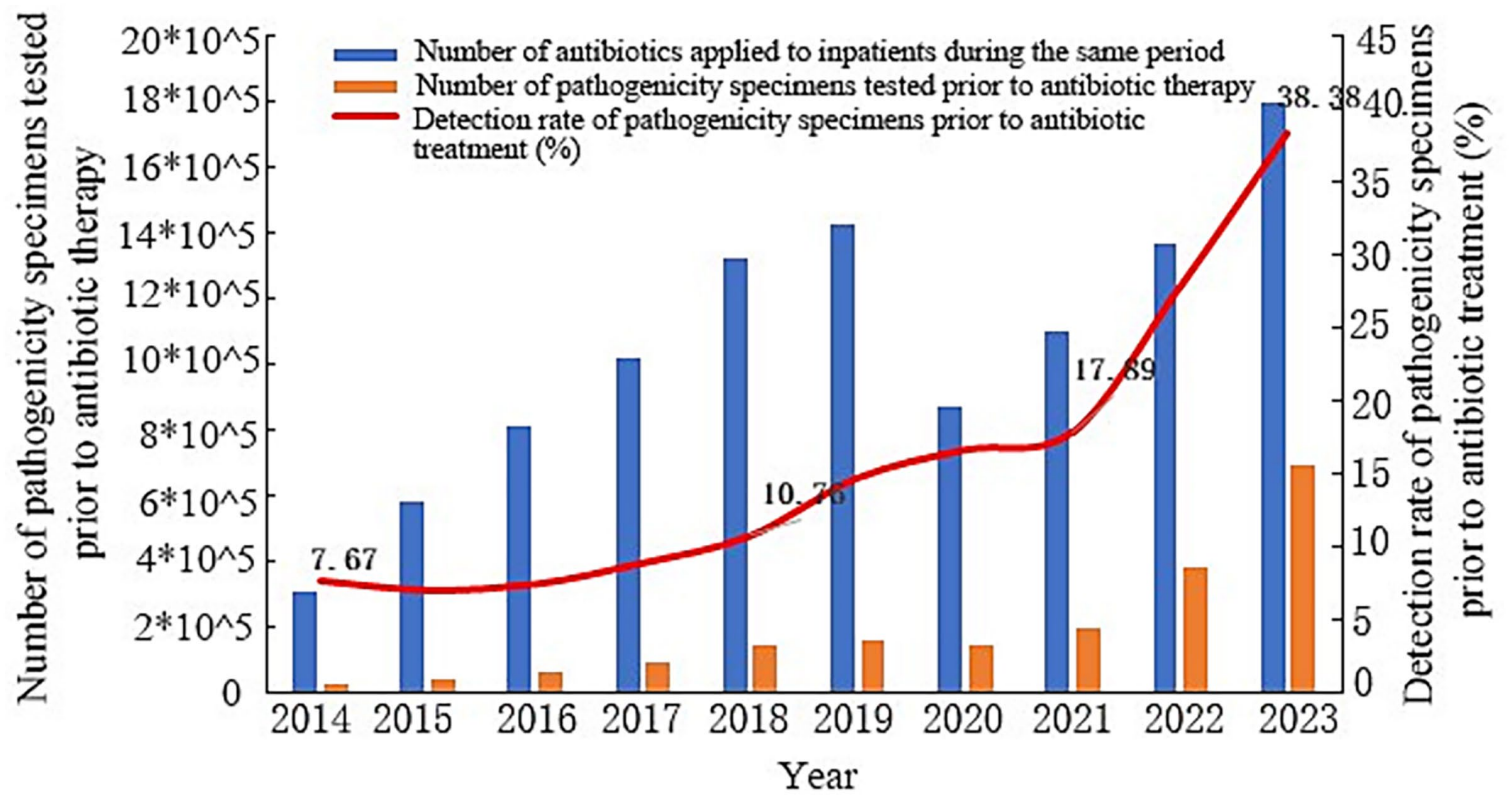
Pathogenicity delivery rate before application of antimicrobial drugs in Shandong Province, 2014–2023 (2023 vs. 2014 **p < 0.01, *χ*^2^ = 108,537.131).

**Figure 3:**
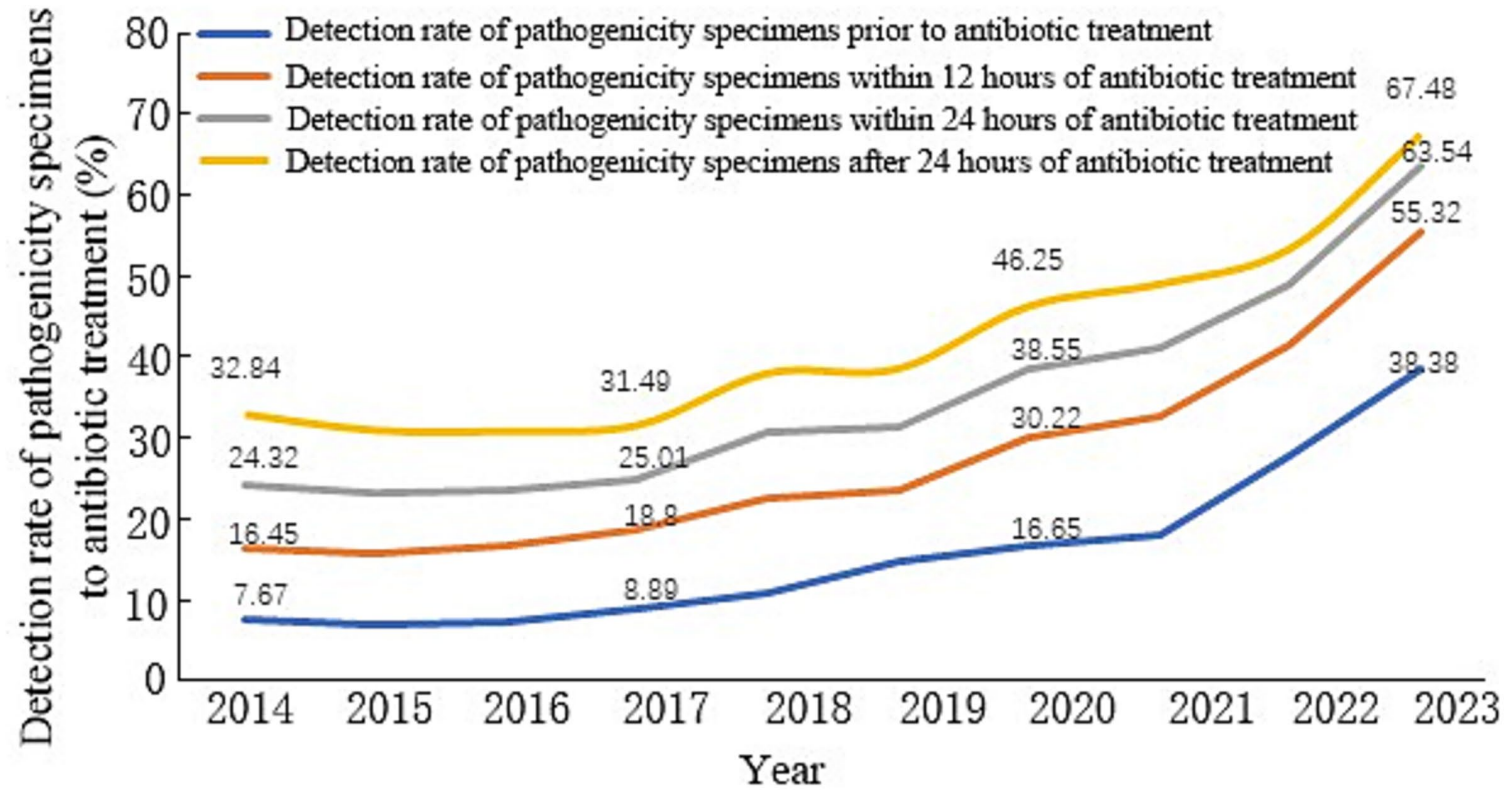
Trend of four pathogenicity delivery rate indicators in Shandong Province, 2014–2023. (**p < 0.01, *χ*^2^ = 132,768.458, 163,555.856, 156,712.142, 108,537.131).

Analysis of data concerning antibiotic treatment duration and specimen submission times within the province indicated an increasing trend in the overall detection rate of pathogenic specimens submitted before antibiotic treatment among hospitalized patients in Shandong Province from 2014 to 2023, rising from 7.67 to 38.38% (*χ*^2^ = 108,537.131, *p* < 0.01, Figure 2). Compared with 2014, the detection rate of pathogenicity specimens within 12 h of antibiotic treatment in Shandong Province in 2023 increased from 16.45 to 55.32% (*p* < 0.01), within 24 h from 24.32 to 63.54% (*p* < 0.01), and before discharge from antibiotic treatment from 32.84 to 67.48% (*p* < 0.01), as shown in Figure 3.

### 2.3 Changes in the detection rate of pathogenicity specimens at different time points of antibiotic treatment

**Figure 4:**
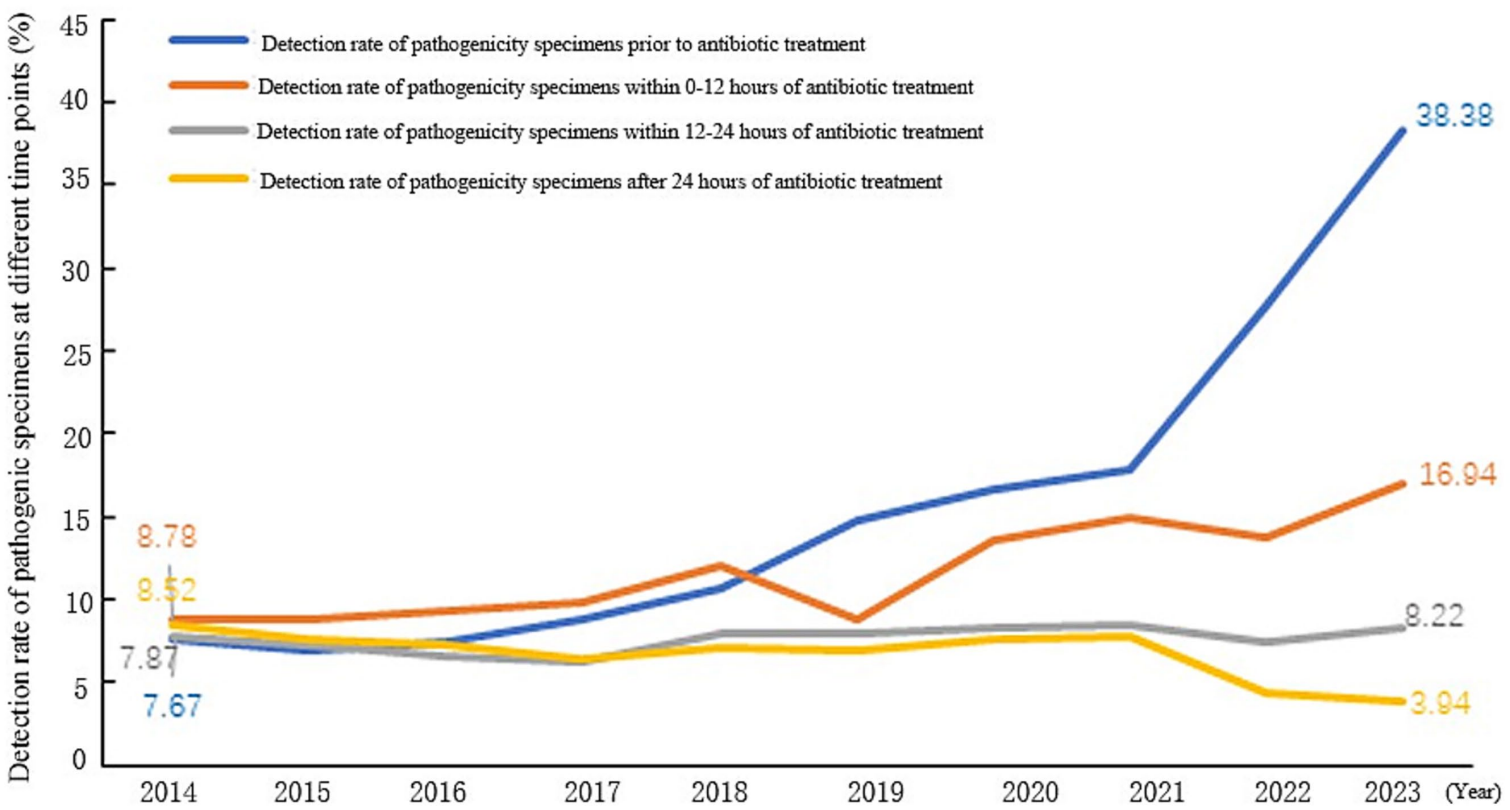
Trend of pathogenicity delivery rate indicators at different time points in Shandong Province, 2014–2023.

Trends in the detection rate of pathogenicity specimens at different time points of antibiotic treatment showed that the detection rate of pathogenicity specimens before antibiotic treatment in Shandong Province increased from 7.67 to 38.38% between 2014 and 2023. A segmented regression model, applied to the data with the intervention point in 2021, revealed that the average annual increase in the detection rate was 1.63% before the intervention, rising significantly to 8.61% afterward (*p* < 0.01). The detection rate of pathogenicity specimens after 0–12 h of antibiotic treatment increased from 8.78 to 16.94%, the detection rate from 12 to 24 h increased from 7.87 to 8.22%, and the detection rate after 24 h decreased from 8.52 to 3.94% (Figure 4).

**Figure 5:**
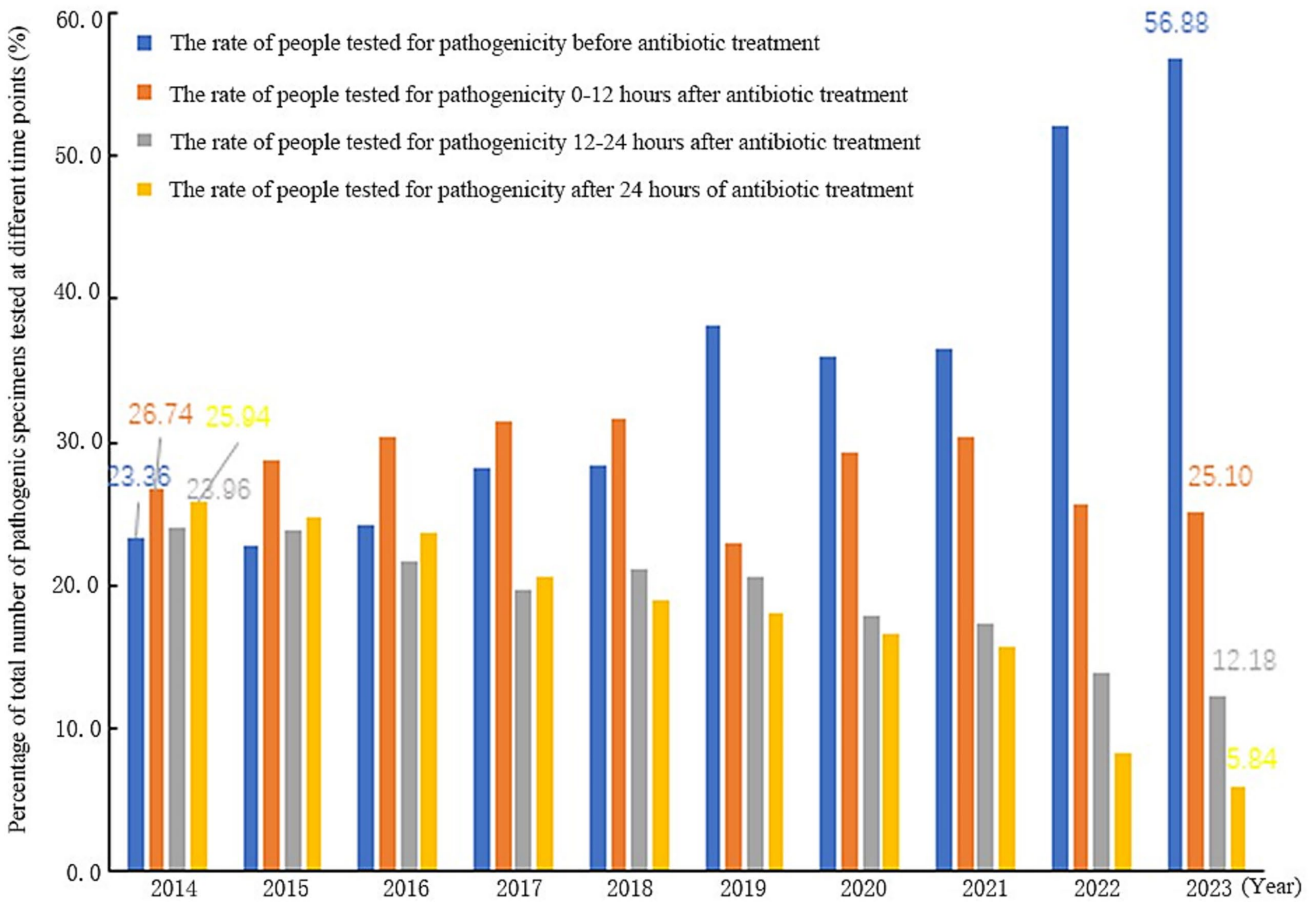
Rate of cases of pathogenicity sent for testing at different points of antimicrobial drug treatment, Shandong Province, 2014–2023.

Figure 5 illustrates the rates of pathogenic specimen submission at different time points relative to the initiation of antibiotic therapy. Comparing 2014 with 2023 in Shandong Province, the proportion of patients whose pathogenicity specimens were collected before antibiotic treatment significantly increased from 23.36 to 56.88%. Conversely, the proportions of specimens collected after antibiotic treatment decreased across all post-treatment timeframes: for collections within 0–12 h, the rate decreased from 26.74 to 25.10%; for collections within 12–24 h, it fell from 23.96 to 12.18%; and for collections after 24 h, it dropped from 25.94 to 5.84%. This indicates that the detection rate of pathogenicity specimens before antibiotic treatment increased significantly from 2014 to 2023, but the number of people with a delay of 12 h was still high.

## 3 Discussion

The use of antibiotics involves all clinical departments and saves countless lives; it is an important cornerstone of modern medicine. However, unnecessary use of antibiotics can lead to serious health problems, and bacterial resistance is one of the risks (9). The rational application of antibiotics is key to improving therapeutic effect, reducing the incidence of adverse reactions, and reducing or delaying the occurrence of bacterial resistance and other adverse health effects. In principle, the selection of antimicrobial agents should be based on the pathogen’s type and its susceptibility to antimicrobial drugs, as determined by antimicrobial susceptibility testing (10). Therefore, healthcare institutions with the necessary facilities should ensure that patients clinically diagnosed with bacterial infections have appropriate, suitable specimens (particularly from sterile sites such as blood) promptly collected and sent for pathogen testing prior to initiating antimicrobial therapy (11). The results of this study showed that the number of healthcare units collecting data increased year by year (to 105 in 2023), and the scope of coverage expanded from seven municipalities to 16 in the province. The increase in the number and scope of units means that the data on the pathogenicity delivery rate are more representative, laying a solid foundation for further improving the province’s pre-treatment pathogenicity delivery rate of antimicrobial drugs.

Pathogen testing conducted before antibiotic treatment emphasizes the critical need for identifying the causative pathogen prior to initiating therapy. The calculation of the indicator requires that the time of specimen collection should be earlier than the time of antibiotic use, and an accurate time of specimen detection and antibiotic use must be obtained. In the ‘Guidelines for Special Action to Improve the Rate of Pathogenesis Delivery and Testing of Inpatients before Antimicrobial Drug Therapy’, it is required that medical institutions should ‘improve the information system related to specimen testing, strengthen the informatization management of medical prescriptions, realize real-time monitoring and early warning, and improve the efficiency of targeted treatment with antimicrobial drugs’ (12). However, at present, there are problems such as inaccurate monitoring data, difficulty in capturing raw data, and low levels of monitoring efficiency and quality in China’s hospital infection informatization construction (13). In clinical practice, empirical treatment without microbiological evidence is frequently employed, which often leads to the selection of inappropriate antibiotics (14). Compared to empirical therapy, treatment guided by bacterial culture tests yields superior outcomes in the management of urinary tract infections. Routine urine cultures should be encouraged to guide the selection of effective antibiotics and reduce antimicrobial resistance (15).

The traditional management of pathogenicity specimen detection for the department is to collect samples, which are then transferred to the laboratory; this management specimen detection efficiency is not high and can easily to lead to antimicrobial drug abuse. At present, 47.37–75.17% (16), hospitals believe that as long as the use of antibiotics for specimen testing can be, statistical indicators without time limits. But such statistics will bias the results. In the results of this study, the traditional way of collecting pre-treatment antibiotic pathogenicity specimen detection rate increased from 32.84 to 67.48%, while the actual pathogenicity specimen detection rate only increased from 7.67 to 38.38% (*p* < 0.01). Therefore, China’s health administration issued the Notice of the General Office of the National Health and Wellness Commission on Continuously Improving the Management of Clinical Application of Antimicrobial Drugs in 2020 and, in 2021 and 2022, further made ‘increasing the rate of pre-treatment pathogenicity testing of antimicrobial drugs in hospitalized patients’ one of the top 10 goals of national healthcare quality and safety improvement (17). Policies and measures require strengthening infection prevention and control, improving the capacity of infection control management, and acknowledging the role of infection control in antimicrobial drug management. Hospitals must also improve information technology construction to help the scientific management of antimicrobial drugs and improve the level of monitoring and analyses. Multi-departmental collaboration mechanisms have been established in different levels of hospitals to strengthen information technology construction, enhance data interconnection and interoperability, carry out MDT discussions, and strengthen the training of medical staff and other comprehensive interventions to accurately collect data and improve the rate of pre-treatment pathogenicity testing of antibiotics, so as to provide an accurate basis for the rational use of drugs in the clinic (18). In addition, this study found that, compared with 2014, the percentage of the number of people tested for pathogenicity specimens after 0–12 h of antibiotic treatment in Shandong Province in 2023 decreased from 26.74 to 25.10%, which suggests that, although the pre-treatment pathogenicity specimens before antibiotic treatment were significantly elevated, the percentage of specimens delayed for 12 h was still high. It is necessary to further increase the intervention for detection within 12 h of antibiotic treatment to further improve the detection rate of pathogenicity specimens before antibiotic treatment.

In the above study, we analyzed the reasons limiting the rate of pre-treatment antibiotic pathogenicity specimen testing: (1) Clinical aspects: a lack of awareness of the rate of pre-treatment antibiotic pathogenicity specimen testing by medical staff, empirical dosing and fluke mentality, and a lack of guidance on reporting and application of guidelines. A survey of 340 physicians and microbiologists in 58 low- and middle-income countries confirmed that guidelines are the most effective antimicrobial stewardship intervention to change clinicians’ antibiotic prescribing behavior. Over a 12-month intervention period, the antibiotic prescribing rate for a decision support tool that provided clinicians with guideline recommendations was 98.7 prescriptions per 1,000 patient-years, compared with 107.6 prescriptions in the usual care group (19). (2) Informatics: one goal of using informatics to support clinical decision-making is to prevent unnecessary antibiotic use (20), and inadequate and untimely information systems similarly constrain the rate of improvement in the testing of antimicrobial pre-treatment pathogenetic specimens. (3) Personnel: bacterial culture programs are not carried out due to long culture times, few sterile specimens, poor implementation of bilateral double vials for blood cultures, and poor local development (21). A study showed that the availability of accessible personnel, testing technology, and other resources affect antimicrobial stewardship (22).

The rational use of antibiotics involves several departments in the hospital, and under the multidisciplinary cooperation model, resources are shared among departments to improve the pathogenicity delivery rate (23). To address the reasons constraining the testing rate of pathogenicity specimens before antibiotic treatment, we set up a project management team integrating administrative management and clinical operation, including functional departments (Infection Management Department, Medical Affairs Department, Nursing Department, and Performance Department), clinical departments (non-compliant departments + poorly executed departments), and safeguard departments (Information Centre, Pharmacy Department, Microbiology Department, and Administration Department). We analyzed the common problems in pathogenicity specimen testing in different levels of medical institutions across the province, provided timely feedback on the problems, and carried out targeted training and MDT case discussions to shorten the time limit for reporting test items in the microbiology room (TAT) to achieve an increase in the rate of antibiotic pathogenicity specimen testing. Dai et al. (24) found that implementing a PDCA (Plan-Do-Check-Act) cycle within a Quality Control Circle’s (QCC) efforts for quality control could increase the inpatient antibiotic pre-treatment pathogenicity specimen detection rate, thereby effectively guiding the rational use of medication in the clinic. Given that staffing constraints compel many hospitals worldwide to rely on conventional workflows, a multi-departmental collaboration mechanism should be established under such circumstances. This would clarify departmental responsibilities and refine the operational protocols of the hospital’s specialized working group for pre-processing antimicrobial drug susceptibility testing and submitting pathogenic specimens. Conducting multidisciplinary team (MDT) discussions and specialized training to implement effective clinical guidance with timely feedback should also be carried out. Training programs should be implemented for healthcare personnel to enhance awareness of pathogen specimen testing, thereby improving detection rates.

In summary, the detection rate of pre-antibiotic therapy pathogenicity specimens in hospitalized patients in Shandong Province from 2014 to 2023 showed an increasing trend, but the delay of 12 h to send specimens for testing needs to be further improved. Systematically integrating research findings on enhancing pathogen testing rates into future antimicrobial stewardship programs hinges on the codification of validated measures into standard operating procedures, complemented by research-data-driven precision training. This transforms the attitude of healthcare professionals from passively complying to actively understanding. Concurrently, establishing a monitoring, feedback, and performance management system centered on submission rates as a core metric will drive continuous improvement. Strengthening multidisciplinary collaboration between infection control departments, microbiology laboratories, and clinical pharmacists to jointly interpret data and formulate strategies will ultimately build a long-term antimicrobial stewardship mechanism transitioning from ‘empirical prescribing’ to ‘precision targeting’. This will enhance healthcare quality and curb bacterial resistance.

## Data Availability

The original contributions presented in the study are included in the article/supplementary material, further inquiries can be directed to the corresponding author/s.
